# Central precocious puberty in girls aged 6 to 8 years and magnetic resonance imaging of the pituitary: 11-year experience in a single centre

**DOI:** 10.1186/1687-9856-2013-S1-P83

**Published:** 2013-10-03

**Authors:** Joanna Yuet-ling Tung, Pik-to Cheung

**Affiliations:** 1 Department of Paediatrics and Adolescent Medicine, Queen Mary Hospital, The University of Hong Kong, Hong Kong

## Introduction

Central precocious puberty (CPP) could be a phenotype of pathology in the central nervous system. While it is generally accepted that all boys with CPP and girls with CPP at less than 6 years of age should undergo brain imaging as part of the workup, there have been controversies as to the use of brain imaging in girls who develop CPP between 6 to 8 years.

## Objectives

To evaluate the prevalence and clinical characteristics of intracranial lesions in patients with central precocious puberty aged 6 to 8 years in a single centre in the past 11 years.

## Methods

Retrospective chart review of girls with CPP and their MRI findings between year 1999 to 2009 in a single centre.

## Results

One hundred and eighty-eight girls had central precocious puberty in the study period and 157 of them (83.5%) had MRI of the pituitary done as part of the workup. The prevalence of intracranial pathology among girls with CPP aged 6 to 8 years was 20.0% while among all girls with CPP aged less than 8 years, 34 girls (21.7%) were found to have intracranial pathology. These pathologies included: pituitary adenoma (n = 16), pineal cyst (n = 8), Rathke’s cleft cysts (n = 4), arachnoid cyst (n = 1), intra-ventricular cyst (n = 1), venous angioma over the left frontal lobe (n = 1), hydrocephalus (n = 2) and an old infarct over the frontal lobe (n = 1). The two cases of hydrocephalus and the case with an old infarct were known before the onset of CPP. None of the lesions detected required further interventions with surgical removal, chemotherapy or radiotherapy within the follow-up period of 7.2 ± 3.0 years.

## Conclusions

Brain imaging the girls with CPP in our centre mainly detected benign lesions not requiring any intervention during our follow-up period. Though the current data do not justify a practice of performing routine MRI for girls diagnosed to have CPP at 6 to 8 years, longer follow-up assessment of such lesions detected in childhood may be necessary before concluding on their benign outcome.

